# SWIVIT - Swiss video-intubation trial evaluating video-laryngoscopes in a simulated difficult airway scenario: study protocol for a multicenter prospective randomized controlled trial in Switzerland

**DOI:** 10.1186/1745-6215-14-94

**Published:** 2013-04-04

**Authors:** Lorenz Theiler, Kristina Hermann, Patrick Schoettker, Georges Savoldelli, Natalie Urwyler, Maren Kleine-Brueggeney, Kristopher L Arheart, Robert Greif

**Affiliations:** 1 University Department of Anesthesiology and Pain Therapy, University Hospital of Bern, Inselspital, Bern, 3010, Switzerland; 2 Anesthesiology Department, University Hospital Center and University of Lausanne, CHUV, Lausanne, 1011, Switzerland; 3 Division of Anesthesiology, University Hospitals of Geneva and Faculty of Medicine, University of Geneva, Geneva, 1211, Switzerland; 4 Division of Biostatistics, Department of Epidemiology and Public Health, University of Miami, Miami, FL, 33136, USA

**Keywords:** Video-laryngoscope, Difficult airway, Airtraq, A. P. Advance, C-MAC, Glidescope, King vision, Mcgrath

## Abstract

**Background:**

Video-laryngoscopes are marketed for intubation in difficult airway management. They provide a better view of the larynx and may facilitate tracheal intubation, but there is no adequately powered study comparing different types of video-laryngoscopes in a difficult airway scenario or in a simulated difficult airway situation.

**Methods/Design:**

The objective of this trial is to evaluate and to compare the clinical performance of three video-laryngoscopes with a guiding channel for intubation (Airtraq™, A. P. Advance™, King Vision™) and three video-laryngoscopes without an integrated tracheal tube guidance (C-MAC™, GlideScope™, McGrath™) in a simulated difficult airway situation in surgical patients. The working hypothesis is that each video-laryngoscope provides at least a 90% first intubation success rate (lower limit of the 95% confidence interval >0.9). It is a prospective, patient-blinded, multicenter, randomized controlled trial in 720 patients who are scheduled for elective surgery under general anesthesia, requiring tracheal intubation at one of the three participating hospitals. A difficult airway will be created using an extrication collar and taping the patients’ head on the operating table to substantially reduce mouth opening and to minimize neck movement. Tracheal intubation will be performed with the help of one of the six devices according to randomization. Insertion success, time necessary for intubation, Cormack-Lehane grade and percentage of glottic opening (POGO) score at laryngoscopy, optimization maneuvers required to aid tracheal intubation, adverse events and technical problems will be recorded. Primary outcome is intubation success at first attempt.

**Discussion:**

We will simulate the difficult airway and evaluate different video-laryngoscopes in this highly realistic and clinically challenging scenario, independently from manufacturers of the devices. Because of the sufficiently powered multicenter design this study will deliver important and cutting-edge results that will help clinicians decide which device to use for intubation of the expected and unexpected difficult airway.

**Trial registration:**

NCT01692535

## Background

Difficult airway management remains a cornerstone of clinical anesthesiology. Difficulty in tracheal intubation is the most common factor related to serious airway complications during general anesthesia [1]. Recently, the combination of the fiberoptic bronchoscope and the laryngoscope led to the development of video-laryngoscopes, providing a video-based view of the glottic opening, with or without additional guidance of the tube towards the tracheal opening. Six devices are under prominent focus in recent publications.

1) The Airtraq (Prodol Meditec SA, Vizcaya, Spain) was the first video intubation device that featured a channel guiding the tube towards the tracheal opening. The blade of the Airtraq is disposable. One study published in 2007 in *Anesthesiology* showed a 100% success rate at first attempt when using manual inline stabilization [2].

2) The A. P. Advance Video-laryngoscope (Venner Medical SA, Singapore) is based on a standard Macintosh laryngoscope that can be used as a stand-alone direct laryngoscope, or as a video-laryngoscope with a monitor attached to the handle and includes a “difficult airway” blade. A manikin study showed short intubation times for certified paramedics with the A.P. Advance [3], but large adequately powered, randomized controlled trials in difficult airway scenarios are lacking.

3) The C-MAC (Karl Storz, Tuttlingen, Germany) features size 2, 3 or 4 Macintosh blades or a “D”-blade (Difficult Airway Blade). The D-blade failed at first attempt in 30% of patients who showed a Cormack-Lehane grade 3 or 4 in a study by the inventor of the design [4]. One study shows a 93% success rate with the C-MAC using size 3 and 4 blades, compared to 84% for direct laryngoscopy when using manual inline stabilization, but intubation took longer [5].

4) The GlideScope (Verathon Inc., Bothell, WA, USA) is a widely used non-guided video-laryngoscope consisting of a curved video blade (single-use or reusable) and a special stylet to be used with the tracheal tube. An observational study in 50 patients showed a 100% success rate of the GlideScope in a difficult airway model using stiff extrication collars [6]. It also reduced intubation times compared with the conventional Macintosh laryngoscope in patients under manual inline stabilization [7].

5) The King Vision (Kingsystems, Noblesville, IN, USA) features either a channeled or a regular, disposable blade size 3. To date, there are no randomized controlled trials available for this device.

6) The McGrath MAC (Aircraft Medical Lt., Edinburgh, UK) is a non-guided video-laryngoscope that features disposable blades. It has been developed from the McGrath Series 5. In a randomized controlled trial with patients with a Mallampati grade of ≥3, the McGrath Series 5 provided a better laryngeal view compared to the C-MAC, but intubation took longer and more intubation attempts where needed [8]. To date, there are no randomized controlled trials available for the McGrath MAC video-laryngoscope.

These optical intubation devices or video-laryngoscopes (VLS) have dramatically improved the quality of glottic visualization. Multiple studies have proven enhanced visibility but not necessarily faster intubation times. Interestingly, in a study on manikins simulating difficult airway with stiff collars, VLS was not superior to direct laryngoscopy, but the sample size was low [9]. Furthermore, while VLS improve visualization of the airway, it is important to realize that a good view of the laryngeal opening does not automatically lead to intubation success. For example, in a recent study, the C-MAC VLS showed a good view of the larynx in 95% of cases, but the actual success rate of the intubation was only 88% [10]. These different success rates cannot directly be compared since these studies were performed in different patient populations by different operators, in different settings regarding difficult airways, and with different outcome parameters. Most importantly, the majority of patients enrolled presented with a normal airway, or only manual inline stabilization was used to simulate a difficult airway. No study compared all these devices in the same setting, and no sufficiently powered study used extrication collars to adequately simulate a clinically important difficult airway situation.

### Specific aims of the study are

1) To investigate which VLS devices reach a clinically acceptable minimal first attempt success rate of 90% in a simulated difficult airway scenario (primary outcome). We assume this lower limit of “90% first attempt success rate” is the lowest tolerable success rate in a difficult airway scenario.

2) To compare primary and overall success rates, view on the tracheal opening and time until intubation with the help of the guided vs. unguided VLS devices.

3) To evaluate possible adverse events, complications and side effects.

According to these specific aims, we propose the following hypotheses:

1) For our primary outcome, we assume that the lower limit of the 95% confidence interval (95% CI) of the first attempt success rate is not lower than 90%. The null hypothesis states that the 95% CI of first attempt success rate is below 0.9.

2) Successful tracheal intubation takes more time using unguided VLS compared with the other VLS devices that guide the tube towards the tracheal opening. The secondary null hypothesis states that there is no statistically significant difference in time until intubation success between a guided and an unguided VLS (two-sided). Other secondary outcome-hypotheses include that the overall attempt success rates are higher in guided VLS compared with unguided VLS.

3) Minor airway injury rates are within a maximum of 10% comparing guided vs. unguided VLS. The null hypothesis states that the differences of minor airway injury rates are higher than 10%.

## Methods/design

### Study design

The SWIVIT trial is a prospective, patient-blinded, multicenter, randomized controlled trial at the anesthesia departments of the University Hospital of Bern, the University Hospital of Lausanne and the University Hospital of Geneva, all in Switzerland.

### Patient population

With ethics committee approval (KEK Bern ref. nr. 106/12 on 11 September 2012; Chairperson: Prof. Dr. N. Tueller) and written informed consent, we will include adult patients of both genders, ASA (American Society of Anesthesiologists) physical status I to III, and scheduled at one of the participating hospitals for elective surgery under general anesthesia requiring tracheal intubation.

Patients are not eligible if they are at risk for aspiration (non-fasted, severe gastro-esophageal reflux disease, hiatal hernia), with known or presumed difficult airways (body mass index >35 kg/m^2^, Mallampati >III, thyromental distance <6 cm, interincisor distance <3.5 cm [11], known difficult mask ventilation or difficult laryngoscopy, or scheduled for awake tracheal intubation), or if they refuse to participate or are unable to give informed consent.

### Sample size calculation

Most previous studies have based sample size calculations on differences of the intubation difficulty score (IDS), developed by Adnet in 1997 for direct laryngoscopy [12]. However, a retrospective study by McElwain has raised concerns about the validity of the IDS with VLS [13]. The most important outcome parameter for VLS or any guided intubation devices is the success rate. Most available data are from patients with normal airway anatomy and, therefore, not comparable with our setting. Only one study immobilized the patients’ necks with collars to investigate the C-MAC in 43 patients. That study found an overall success rate of 88% [10]. This is an unacceptable low success rate for the management of a difficult airway, but the study was underpowered: we calculated the 95% CI in that study to be 0.75 to 0.95 for overall success rate.

In order to rate an intubation device as “successful”, we define that the lower limit of the 95% CI should not be smaller than 0.9. We based our sample size on these values, congruent with our findings in a small pilot sample. We calculated the necessary sample size to obtain a distance of 0.05 to the expected success rate of 0.95, provided a probability of 0.95 and a power of 80% (SAS v.9.1, SAS Institute Inc., Cary, NC, USA). A total of 107 patients per device are necessary for this lower limit of 0.9. A total of 642 (6 × 107) patients will be necessary based on these assumptions, which leads us to include 720 patients to compensate for dropouts or missing data.

Secondary endpoints include parametric data, such as time necessary until success. Time until success has varied widely among different devices in published studies. Furthermore, that parameter seems to be influenced by the anesthesia provider. Therefore, we based our sample size calculation on first attempt success rate.

Because available data about our primary outcome, the first attempt success rate in a simulated difficult airway scenario, are scarce, we will recalculate sample size after the first 120 patients, based upon the values obtained from the first 20 patients for each device. In order to reduce bias, all participating investigators will remain unaware of results obtained from these 120 patients.

We will compare time to intubate in unguided vs. guided VLS as the secondary outcome. There are not sufficient data available to calculate sample size for this secondary outcome parameter. Therefore, we base our sample size calculation on effect size: based on an estimated medium effect size (Cohen’s d of 0.5; assuming normal distribution), 64 samples per group are necessary, given a 0.05 level (*P* = 0.05) and 80% power. Because our primary outcome requires 107 patients per group, we are well within the necessary sample size even for this secondary outcome parameter.

Experience with the device is expected to be a major confounding factor. Only a limited number of experienced anesthesiologists at each study center will perform the intubation. To avoid a learning curve bias and to minimize variation in the performance, only anesthesiologists who have intubated patients without any airway pathology several times with each device until they feel competent with the devices will participate. We did not set *a priori* a fixed figure of intubations for each device because the individual experience with different devices is very divergent between the study centers and the participating anesthesiologists. Ideally, experience with the device should be equal, and prior experience will be recorded. We limit participants per center to a maximum of four physicians.

### Statistical analysis

For the primary outcome, it will be analyzed for each laryngoscope whether the 95% confidence interval of its primary success is below 0.9. For the secondary outcome parameters, the data distribution will determine which statistical test will be used. For frequencies (for example, number of required manipulations, overall attempt success, complications) chi-square test or Fisher’s exact test will be used. Parametric data will be analyzed using ANOVA; for non-parametric continuous data Kruskal-Wallis test will be used. For comparison between two devices, we will use Student’s *t*-test and Mann-Whitney’s *u*-test, as appropriate. We will do *a priori* comparisons of the time necessary until success between the unguided VLS and any of the guided VLS.

Data will be presented as mean with standard deviation, median and interquartile range, or number and percent. Effect sizes (with 95% CI) will be reported as Cohen’s d for interval data and as odds ratio for proportions. A probability of 0.05 is considered as statistically significant.

**Figure 1:**
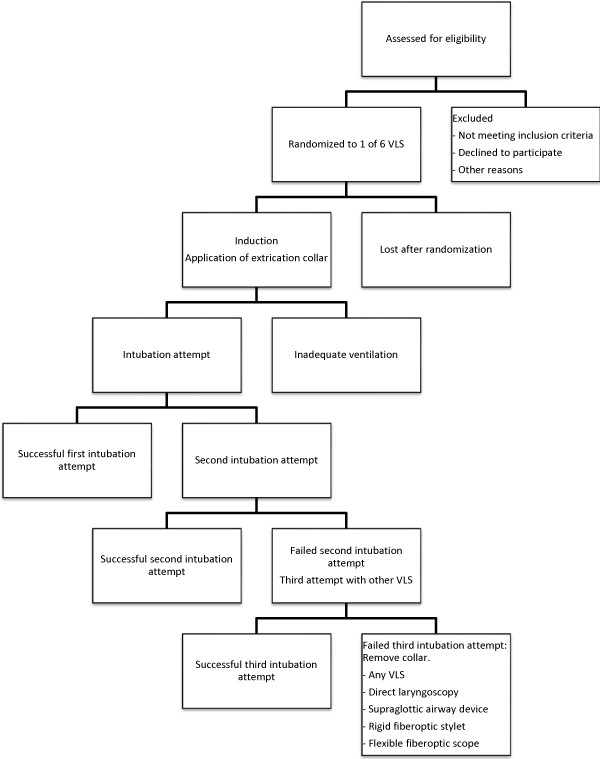
CONSORT flow diagram.

The patient flow diagram according CONSORT guidelines is provided in Figure 1.

### Detailed study plan

#### Consent procedure

Patients will be recruited from the operating room schedule of the corresponding hospitals. Written, informed consent will be obtained from each patient on the day before surgery. All participants will be given a copy of the *Patient Information Sheet* and be specifically informed that they may decline to participate in or withdraw from the study at any time.

#### Allocation of patients

Patients with written informed consent are randomly allocated to one of the six devices. The allocation sequence will be generated using online randomization software (http://randomization.com) in blocks of 30 intubations for the devices and stratified for each participating center and for each physician. The allocation will be concealed in sealed, opaque, sequentially numbered envelopes and will not be opened until the patient is anesthetized.

#### Clinical study procedure

Premedication will be according to the standard operating procedures of the participating centers. Standard non-invasive monitoring includes ECG, non-invasive arterial blood pressure, oxygen saturation (SpO_2_), end-tidal CO_2_ and volatile anesthetic level if applicable. A bispectral index (BIS, Aspect Medical Systems, Norwood, MA, USA) or a different processed EEG monitoring will be used whenever available. Anesthesia will be induced with propofol 1.5 to 3 mg/kg body weight and fentanyl 1 to 2 mcg/kg body weight. After facemask ventilation is established, neuromuscular blocking agents (rocuronium 0.6 mg/kg body weight) will be given and appropriate action monitored by neuro-stimulation. The inter-incisor distance at maximum mouth opening will be measured while the patient is asleep. Then, the extrication collar (Stifneck™, Laerdal, Copenhagen, Denmark) will be properly adjusted, and a self-adhesive tape will be used to fix the head on the operating table, as done in an earlier study by our group [6]. The inter-incisor distance will be measured at maximal mouth opening aiming at a mouth opening between 20 to 25 mm.

If mask ventilation remains adequate and a sufficient level of anesthesia is confirmed (BIS <55, stable hemodynamic parameters, unresponsiveness to jaw thrust), the tracheal intubation will be performed with the help of one of the six VLS, according to randomization.

#### Selection of tracheal tube size (Mallinckrodt Hi-Contour Oral/Nasal Tracheal Tube Cuffed, Covidien, Hazelwood, MO, USA)

Women: 6.5 mm ID (internal diameter)

Men: 7.5 mm ID

#### Selection of blade size

1) Airtraq: Size #2 in women (6.5 mm tracheal tube does not fit in size #3 device), size #3 in men

2) A. P. Advance: Difficult airway blade for guided intubation

3) C-MAC: D-blade. Additionally, a pre-shaped stylet (shaped according to the decision of the consultant anesthesiologist) will be used for tracheal insertion.

4) GlideScope: GVL single use blade #3 with reusable GlideScope stylet for tracheal tube

5) King Vision: Blade #3 (channeled)

6) McGrath MAC: Disposable McGrath MAC blade. Additionally, a pre-shaped stylet (shaped according to the decision of the consultant anesthesiologist) will be used for tracheal insertion.

The primary endpoint is successful tracheal intubation confirmed by capnography (CO_2_ monitoring). Further management of anesthesia is according to the consultant anesthesiologist.

#### Device failure

A device failure is defined as two unsuccessful intubation attempts with a maximum of 180 seconds for each attempt while oxygen saturation remains >90%. The intubation attempt is allowed to continue if the laryngeal opening is identified after 180 seconds and the patient does not desaturate (SpO_2_ >90%). However, this will not count as success at the first attempt, but as an overall attempt success. After the second unsuccessful attempt, a third and last attempt will be performed with another device, chosen according to the decision of the attending anesthesiologist with the rigid collar in place. In case of failure of the second device, further airway management will be according to the decision of the attending anesthesiologist, and without the rigid collar.

#### Break-up criteria (leading to removal of the rigid collar)

Primary and secondary VLS device failure

Bronchospasm, injury

Technical failure of the intubation devices during insertion attempt (for example, light bulb failure or monitor failure)

If a break-up criterion is reached, the extrication collar will be removed, the patient ventilated if necessary and the trachea will be intubated via either the randomized device (one attempt) or any other further airway management device, according to the attending anesthesiologist. The attending anesthesiologist may choose another airway management strategy once a break-up criterion is reached.

#### Measurements

Insertion success (first and second attempt success rate).

#### Definition of success

Lung ventilation through the cuffed tracheal tube, confirmed by end-tidal CO_2_.

Time necessary for completion of the first attempt intubation, second attempt (if applicable) and overall intubation (calculated as the sum of the first and, if applicable, second attempt).

#### Definition of time

Time necessary until success is measured from the time the facemask is taken away from the face until the end-tidal CO_2_ curve appears on the monitor of the respirator. Time for each attempt is measured separately.

Cormack-Lehane (CL) grade [14] at laryngoscopy (not developed for indirect laryngoscopy, but necessary for the calculation of the IDS).

Inter-incisor distance before induction of anesthesia and after placement of the collar to measure maximum mouth opening and the reduction of mouth opening by the collar (collar adjusted to permit a minimal opening of 18 mm, according to the minimal requirements for Airtraq and King Vision)

Number of optimization maneuvers required (cricoid pressure or BURP, backward, upward, rightward pressure), second assistant, adjustment of head positioning) to aid tracheal intubation [15].

View on the glottic opening in percent (%) as judged by the operator: Percentage of Glottic Opening (POGO) Score [16].

Adverse events: cardiovascular extremes: any hypo-/ hypertension and tachycardia/ bradycardia exceeding 20% from baseline. Blood on device, and injury during intubation attempt, suspicion of aspiration/regurgitation (gastric fluid in the ventilation tube or in the hypopharynx), hypoxia (SpO_2_ <90%), bronchospasm, airway obstruction or any other form of stridor, coughing, dental, tongue or lip trauma.

Technical problems with the device, such as fogging, impeded vision and monitor/light source failure.

The Intubation Difficulty Scale (IDS) score will be calculated as previously published [12], using the following parameters: number of attempts and operators, alternative techniques, Cormack-Lehane grade, lifting force, laryngeal pressure and vocal cord mobility.

Demographic and perioperative data: sex, age, weight and body mass index, dentition, surgical procedure/ duration, duration of anesthesia, date/ time of hospital discharge.

#### Postoperative evaluation by blinded personnel

After anesthesia, the recovery room nurse (blinded about randomization) will use a checklist to assess for any airway trauma. The checklist, which includes questions about active oral bleeding, coughing blood, blood-stained saliva, sore throat, pain when swallowing, coughing, postoperative nausea and hoarseness, follows a 3-graded assessment by the patient (mild, moderate, severe). Timing of this assessment will be standardized to one hour after post-anesthesia care unit admission. If patients are extubated in the intensive care unit (ICU), the ICU nurse will make the assessments. On postoperative day one, a member of the study personnel will make a further assessment, using the same checklist. In case of ambulatory surgery, assessment will be done by telephone. The investigator will be unaware of the randomization, any problems encountered during intubation or surgery, and will be blinded about the performance of the airway device.

#### Data collection techniques

All clinical data will be collected by a research assistant at bedside, using digital data recording devices (tablets). In case of device failure, a back-up paper form will be available. All data will be sent to a secure, central data storage immediately after the closure of the local case report form.

#### Ethical approval

The SWIVIT randomized controlled trial has been approved by the ethic committee of the canton of Bern (KEK Bern ref. nr. 106/12 on 11 September 2012).

**Figure 2:**
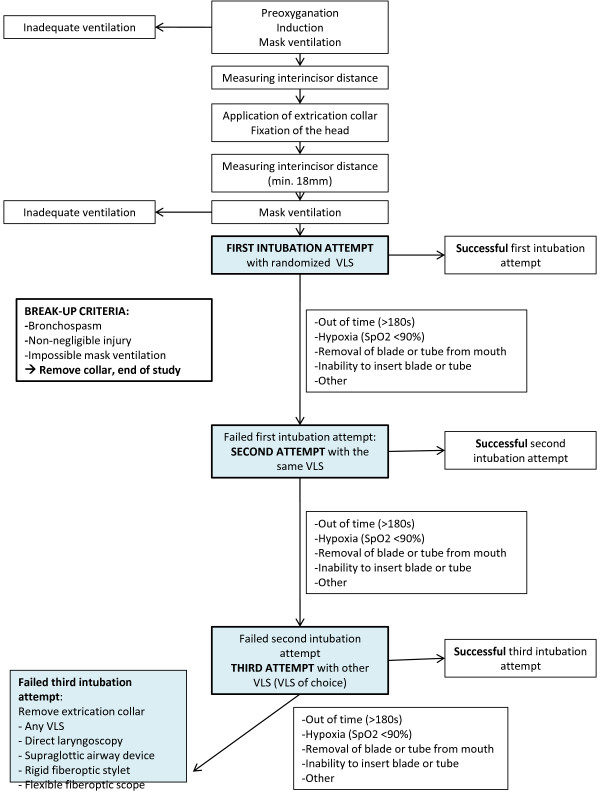
Study flow chart.

A summarizing study flow chart is provided in Figure 2.

## Discussion

According to the latest national audit in anesthesia in the UK, major adverse events are estimated to be as high as 1 in 5,500 anesthesia cases, leading to brain damage and even death [1]. Airway management was deemed to be good in only 19% of these cases. This is an unacceptable high failure rate for our patients’ safety. Therefore, new and supposedly better devices to manage the difficult airway are necessary and continue to enter the market, often without thorough evaluation of their efficacy. In case of use of such devices in a difficult airway situation, it is vital to know which device will perform best. While VLS are marketed to facilitate the tracheal intubation in difficult airway management, there are no adequately powered data available comparing VLS in real difficult airway situations or, at least, adequately simulated difficult airway models. We intend to deliver this evidence. This will be for the benefit and safety of patients presenting with an expected or unexpected difficult airway needing general anesthesia for surgery or interventions.

### Potential problems and limitations

Because sample size has been calculated based on relatively vague figures, some adjustments in the total, necessary sample size may have to be incorporated when we re-calculate our sample size after the first 120 patients as per protocol. However, the statistical calculation is sound, and our assumptions are based on highly probable clinical expectations. Furthermore, the results of our study will be of clinical relevance regardless of whether our primary hypothesis will be confirmed or not and the much-feared “negative results” should be of no concerns in this study.

### Generalizability

In this clinical trial, we will use a statistical model that may be incorporated in future trials as well. Most clinical airway studies seek to prove a difference between devices, or postulate agreement within pre-defined values, some are designed as so called “non-inferiority” trails. In this study, we pre-define an important clinical value as a benchmark on which all devices studied are compared with the 90% minimal first attempt success rate. We believe an airway device should strive for this success rate, although that would still mean a failure in 1 out of 10. However, standard procedures in the difficult airway model used for this study have been shown to perform even less well: direct laryngoscopy succeeds only in 39.5% of cases [17] and rigid fiberoptics fail in 9 to 14% (own data, not published yet). Flexible fiberoptic intubation would be the method of choice, however, it is highly operator dependent and time consuming [18], and showed recently a not that impressive first attempt success rate of only 79% [19].

## Trial status

At the time of submission, the study was actively enrolling patients. Fewer than 10 patients had been enrolled in total in all three centers.

## Abbreviations

ASA: American Society of Anesthesiologists; BIS: Bispectral index; BURP: Backward, upward, rightward pressure; CI: Confidence interval; CL: Cormack-Lehane; ECG: Electrocardiography or electrocardiogram; EEG: Electroencephalography or electroencephalogram; ID: Internal diameter; IDS: Intubation Difficulty Scale; POGO Score: Percentage of glottic opening score; SpO2: Oxygen saturation; SWIVIT: Swiss video-intubation trial; VLS: Video-laryngoscopes

## Competing interests

We received the Airtraq™ from Prodol Meditec SA represented by MK-MED AG in Switzerland, the A. P. Advance™ from Venner Medical SA, the C-MAC™ from Karl Storz represented by Anklin in Switzerland, the Glidescope™ from Verathon Medical Inc. represented by Anandic Medical Systems in Switzerland, the King Vision™ from Kingsystems represented by Anel GmbH in Switzerland, and the McGrath™ from Aircraft Medical Lt. represented by Covidien in Switzerland, all without costs. The authors declare that they have no competing interests.

## Authors’ contributions

RG and LT are the principle investigators of this study, both developed the study design and drafted and revised the protocol. KH contributed to the preparation of the study, the clinical report form, and the final protocol, designed the first draft of the manuscript, and is responsible for the organization of data acquisition as well as the coordination among the three centers (study coordinator). NU and MKB assisted in the development of the protocol, and in the final writing and reviewing of the manuscript. PS is the local principle investigator at the University Hospital of Lausanne, contributed to the final protocol and oversees the study project at that site. GS is the local principle investigator at the University Hospital of Geneva, contributed to the final protocol and oversees the study project at that site. KA helped in establishing the protocol and contributed the statistical calculations. All authors read and approved the final manuscript.
